# The role of fear of intimacy and loneliness in predicting of academic enthusiasm in medical science students: a cross-sectional study from the Iran

**DOI:** 10.1007/s44192-025-00257-8

**Published:** 2025-07-28

**Authors:** Mohammad Reza Asadi, Mojtaba Senmar, Zahra Shafiei kisomi, Zahra Hosseinkhani, Amir Ehsan Ahmadi, Mohammad Mozafari, Faraz Oustam, Ali Ahmadieh

**Affiliations:** 1 Student Research Committee, Qazvin University of Medical Sciences, Qazvin, Iran; 2 Non-Communicable Diseases Research Center, Research Institute for Prevention of Non-Communicable Diseases, Qazvin University of Medical Sciences, Qazvin, Iran; 3 Social Determinants of Health Research Center, Research Institute for Prevention of Non-Communicable Diseases, Qazvin University of Medical Sciences, Qazvin, Iran; 4 Department of Medical-Surgical Nursing, School of Nursing and Midwifery, Tehran University of Medical Sciences, Tehran, Iran

**Keywords:** Academia, Fear, Loneliness, Students, Medical

## Abstract

**Background:**

Academic enthusiasm is one of the most effective factors in achieving the best in students’ education. Therefore, this study aimed to determine the role of fear of intimacy and loneliness in predicting academic enthusiasm in medical science students. this descriptive-cross-sectional study was conducted among students of Qazvin University of Medical Sciences in iran, 2023–2024. The data was collected using the demographic profile checklist, Frederick et al.’s academic enthusiasm inventory, Russell’s loneliness scale, and Thelen and Descunter's fear of intimacy scale. The data was analyzed using descriptive-analytical statistics and SPSS version 22 software.

**Results:**

In total, 212 (55.5%) of the 380 students participating in the study were female and the rest were male. The mean age of the students was (21.83 ± 9.97) years. According to the results, students’ academic enthusiasm with an average of 42.79 ± 8.09, and a score of 45.0 out of 100 is average. Based on the results, the feeling of loneliness with a standard score of 45.0 and a mean of 47.02 ± 9.76, and fear of intimacy, with a standard score of 40.86 and a mean of 92.21 ± 20.17, are at the moderate and below-average levels, respectively. The results of univariate regression analysis showed that for one unit increase in loneliness score, the academic enthusiasm in students decreased by 0.17 (*P* < 0.001).

**Conclusion:**

The results showed that in medical science students, academic enthusiasm and loneliness are at an average level, but the fear of intimacy in this group is below the average level. In addition, the results showed that the feeling of loneliness has a predictive role in academic enthusiasm, and as the feeling of loneliness increases, academic enthusiasm decreases. Based on this, students are expected to participate in cultural workshops, communication management and motivation to reduce feelings of loneliness and promote academic enthusiasm.

## Background

Medical students have a special importance in improving the quality of healthcare services in a country [1]. In education, especially medical sciences, to reach the optimum, all effective factors must be considered to solve educational problems and improve the health of society by using them optimally [2]. One of these factors is the academic enthusiasm of students [2].

Academic enthusiasm refers to a person’s internal desire that direct his behavior towards learning and academic progress [3, 4]. This construct refers to the amount of time and energy that learners spend intentionally and planned on educational and learning activities [5]. People with academic enthusiasm perform better and show more commitment to rules and duties [6]. This educational structure as a motivational structure [5], according to the latest statistics obtained in studies inside Iran, has obtained a score of 46.66% [7]. At the same time, the provision of high-quality care services requires students with sufficient enthusiasm to receive the required information and skills [8, 9] and in the fields of medical sciences that deal with human lives, the lack of enthusiasm has destructive effects on the individual and the society [3]. Although there is a high rate of dropping out among students with low academic enthusiasm [10], this protective factor against challenges and problems [6] is influenced by personal resources, perception, efficacy, and social resources or social support. [11–14]. Considering the effect of social support on academic enthusiasm, fear of intimacy as a social factor may be effective on academic enthusiasm.

Fear of intimacy is expressed by a person’s limited capacity to share personal thoughts and feelings with close and important people [15]. When a person’s capacity and ability to express intimacy is depleted, fear of intimacy is formed [16]. People with a high level of fear of intimacy not only have difficulty in creating and maintaining satisfactory relationships [17], but fear of their intimacy can also cause anxiety social phobia, and lack of self-confidence [18]. In the search conducted, no similar study was found that measured the relationship between fear of intimacy and academic enthusiasm. Factors such as participation with peers, interaction with faculty members, academic support, and peer support can lead to enthusiasm in students [19]. Studies conducted in the field of academic enthusiasm also emphasize the environment and external factors such as the quality of the relationship with parents, the emotional atmosphere of the family, and the quality of interaction with the teaching staff and professors [20]. Another predictor of students’ participation and academic performance is the feeling of loneliness [21].

Loneliness with different emotional and social dimensions, affects different age groups [22, 23]. Loneliness refers to the difference between expected and received relationships [21]. This negative subjective experience occurs when social relationships and interactions are considered insufficient [24]. Due to various challenges and issues during their studies, medical students are prone to psychological and emotional problems that can lead to academic failure. One of these problems is feeling alone [25, 26]. Approximately one-third to one-half of students experience loneliness during their university years [27]. Research indicates that loneliness is prevalent within the medical health industry [28]. Statistics also show that 50.5% of medical students experience mild loneliness and 31.6% experience severe loneliness [25]. the evidence shows that loneliness, as a strong precursor, can intensify stress, depression, anxiety, and suicide [29, 30] and has a direct relationship with the lifestyle and behavior of medical students [25, 31]. Therefore, loneliness is not only a risk factor for negative health outcomes but it is also associated with a high risk of academic failure [32]. In the search performed no similar study was found that dealt with the relationship between loneliness and academic enthusiasm.

The student period is one of the periods in which people have many concerns [33]. Medical students will form the main form of the future health system of society [34]. In education, especially in medical sciences, to reach the optimum level, all effective factors must be considered to improve the health of society, one of these factors is academic enthusiasm [2]. Academic enthusiasm among these students causes more effective learning of clinical skills and theoretical knowledge [7]. In addition, this concept, it can be a solution to deal with academic issues and challenges [6]. It is believed that the educational goals are achieved only when the students play their role properly. Therefore, it should be said that better performance and motivation depend on enthusiastic students [35]. But, the social, cultural, and economic conditions of society affect many students and and expose them to harm and moral and behavioral abnormalities [25]. Among these injuries can be loneliness and fear of intimacy. Variables that can affect students’ performance and academic enthusiasm. Despite the importance of these categories in medical students, few studies have been done in this field and limited data is available. Therefore, the present study was conducted to determine the predictive role of fear of intimacy and loneliness in the academic enthusiasm of medical science students.

## Methods

### Setting and study design

The present descriptive-cross-sectional study was conducted among students of Qazvin University of Medical Sciences, Iran in 2023–2024. Considering 1μ = 1.4, 1δ = 0.58, 2μ = 1.53, 1δ = 0.62, α = 0.05, and β = 0.80, the sample size was 336 people, and including 15% attrition, 380 people were included in the study.

$$ n = \frac{{\left( {z_{1} - \frac{\alpha }{2} + z_{1} - \beta } \right)^{2} \left( {\sigma_{1}^{2} + \sigma_{2}^{2} } \right)}}{{\mu_{1} - \mu_{2} }} $$

According to the ratio of students of each faculty to the total number of university students, 186 people from the nursing college, 57 people from the health college, 59 people from the paramedical college, and 78 people from the college of dentistry and medicine were included in the study. Due to variations in class schedules, students were present on campus at different times. Consequently, participants were selected using convenience sampling. Willingness to participate in the study, age over 18 years, passing at least one academic semester, not being employed full-time and not having a psychological illness according to the person’s statement were the criteria for entering the study. Incomplete completion of the questionnaires led to sample withdrawal from the study. Prior to initiating data collection, official permission to conduct the study was obtained from the administrative authorities of each faculty. Participants were selected in person after visiting the respective faculties to complete the questionnaires. The study objectives were clearly explained to each individual. Participants were assured that their data would remain strictly confidential. The questions were read to participants, and their responses were recorded. Students were allowed to leave the study at any stage they wanted or not to answer some questions. No attrition occurred during the study, and all collected questionnaires were included in the final analysis.

### Data collection tools

#### Demographic checklist

This checklist included age, sex, marital status, place of residence, academic semester, grade point average, College, birth rank, and student work history.

#### Academic enthusiasm inventory

This questionnaire was created by Frederick et al.’s (2004). This questionnaire has 15 items and measures academic enthusiasm with a five-point Likert scale (1-never, 2 = rarely, 3 = sometimes, 4 = most of the time, and 5 = always). In this questionnaire, which has three behavioral, emotional, and cognitive subscales, scores between 15 and 25 indicate low academic enthusiasm, 25–50 indicate moderate academic enthusiasm, and scores above 50 indicate high academic enthusiasm [2]. The designers of the scale have reported the reliability coefficient of this scale as 0.86. The validity and reliability of the tool have been confirmed in Iranian society [2, 36].

#### Fear of intimacy scale

This 35-item scale is self-reported and prepared by Thelen and Descunter [37]. Subjects are asked to complete the questionnaire while imagining that they are in a close relationship and rate each of the 35 items on a 5-point scale ranging from 1 (does not describe me at all) to 5 (does not describe me at all). Items 3, 6, 7, 8, 10, 14, 17, 18, 19, 21, 22, 25, 27, 29, and 30 are reverse scored. The total possible scores range from 35 to 175. The findings of thelen and descunter show that people who score high on this scale have many problems in the field of intimacy [38]. In Iran, Pourehsan and his colleagues obtained Cronbach’s alpha of 0.85%, and its validity was also confirmed by professors of Shahid Bahonar University of Kerman [38].

#### Loneliness scale

The Loneliness Questionnaire was created by Russell et al. in 1980. This 20-question questionnaire has 10 negative and 10 positive sentences. Answers to the questions are on a four-point Likert scale (never (1), rarely (2), sometimes (3) and always (4)). The range of scores is between a minimum of 20 and a maximum of 80. A score between 20 and 34 indicates mild loneliness, 35–48 indicates moderate loneliness, and above 48 indicates severe loneliness [25]. The reliability of the test was reported by Russell to be 0.89 [39]. The validity and reliability of the tool have been confirmed in Iranian society [25].

### Statistical analysis

After entering the data into the software and cleaning it, the normality of the data was checked using the Kolmogorov–Smirnov test. Tests such as mean and standard deviation were used to describe quantitative variables, and frequency and percentage were used to describe qualitative/nominal variables. To investigate the relationship between the variables, first, univariate regression tests were used; Then, to adjust the effect of confounding variables, multivariate regressions were used. The skewness-kurtosis test were performed (Skewness = 0.142(0.12),Kurtosis = 0.030(0.25)), which showed that the data were normal. Durbin-Watson coefficient was also 1.88. The significance level was considered 0.05. Analyzes were performed using SPSS version 22 software.

## Results

In total, out of 380 samples included in the study, 212 (55.5%) were female and the rest were male. Most of the participants were single (96.1%). The mean age of the participants in the study was 21.83 ± 9.97 years. Nearly half of the participants were nursing faculty students (48.7%). The mean grade point average of the students was 16.39 ± 1.90 (Table 1).

**Table 1:** Demographic characteristics of students participating in the study (n = 380)

Variable		N (%)
Academic semester	≤ 3	156(40.8)
	4–5	89(23.3)
	6–7	114(29.8)
	8 ≤	23(6.0)
Birth rank	1	188(49.2)
	2	111(29.1)
	3	81(21.2)
Marital status	Single	(96.1) 365
	Married	(3.9) 15
Place of residence	Private house	(51.1) 194
	University dormitory	(48.9) 186
Student work history	Yes	(25.3) 96
	No	(74.7) 284

To reduce the comparison error and ease the comparison of the scores of the 3 main variables, standardization of 100 scores was done. According to the results, students’ academic enthusiasm with a mean of 42.79 ± 8.09, and a score of 45.0 out of 100 is average. The highest score in this tool was related to the behavioral academic enthusiasm subscale with a standard score of 50.19 out of 100 (mean = 1.89 ± 12.03). The results showed that the fear of intimacy with a standard score of 40.86 out of 100 (mean = 92.21 ± 20.17) is below average. In the separate evaluation of the fear of intimacy scale, the highest score was related to the subscale of fear of intimacy with others with a standard score of 46.10 out of 100 (mean = 14.22 ± 4.46). In addition, the results showed that the feeling of loneliness with a standard score of 45 out of 100 (mean = 47.02 ± 9.76), is at the average level (Table 2).

**Table 2:** Mean (± Sd) academic enthusiasm, fear of intimacy and loneliness in students

Variable		Mean (± Sd)	Mean (score out of 100)
Fear of intimacy	In the relationship with the spouse	77.99(17.91)	40.00
	In the relationship with the others	14.22(4.46)	46.10
	Total	40.86	92.21(20.17)
Academic enthusiasm	Behavioral	12.03(1.89)	50.19
	Emotional	17.30(4.26)	47.08
	Cognitive	13.46(4.26)	42.30
	Total	42.79(8.09)	46.32
Loneliness	Total	(9.76)47.02	45.0

The results of univariate regression analysis showed that for one unit increase in loneliness score, students’ academic enthusiasm decreased by 0.17. After adjusting the effect of confounding variables, this rate reached 0.16 and the relationship was significant (*P* < 0.001). Fear of intimacy did not show a significant relationship with students’ academic enthusiasm. According to the results of univariate regression analysis, for one unit increase in students’ Grade point average, their academic enthusiasm increased by 0.80, which reached 0.71 in multivariate analysis and the relationship was significant (*P* = 0.001). The results of multivariate regression showed that the academic enthusiasm between male and female students has a significant difference (*P* = 0.016). In the students who were in the 6th–7th semesters, compared to the students in the 3rd and lower semesters, for one unit increase in the academic semester, academic enthusiasm decreased by 3.53 units (*P* < 0.001). In other academic semesters, this difference was not significant (Table 3).

**Table 3:** Regression analysis of predictors of academic enthusiasm in students

Variable	Multiple	Uni -variate
	β coefficient (95% CI) *p* value	β coefficient (95% CI) *p* value
Fear of intimacy	–	**− **0.03(**− **0.07, 0.004) *P* = 0.085
Loneliness	**− 0.16(-0.24**, **− 0.08)** ***P* < 0.001**	**− 0.17(− 0.25**, **− 0.09)** ***P* < 0.001**
Age		**− **0.02(**− **0.10, 0.06) *P* = 0.647
Grade point average	**0.71(0.30**, **1.11)** ***P* = 0.001**	**0.80(0.37**, **1.22)** ***P* < 0.001**
*Sex*		
Female	1	1
Men	**− 1.19(− 2.16**, **− 0.22)** ***P* = 0.016**	**− **0.64(**− **2.26, 1.02) *P* = 0.459
*Academic semester*		
3 ≥	1	1
4–5	**− **1.48(**− **48, 0.52) *P* = 0.146	**− **1.67(**− **3.74, 0.39) *P* = 0.113
6–7	**− 3.53(− 5.39**, **− 1.67)** ***P* < 0.001**	**− 3.94(− 5.86**, **− 2.02)** ***P* < 0.001**
8 ≤	1.12(**− **2.36, 4.61) *P* = 0.529	0.97(**− **2.35, 4.59) *P* = 0.598

Significant variables are bolded

## Discussion

### Academic enthusiasm

The present study showed that academic enthusiasm in students is at an average level and below average. This finding is in line with some Iranian studies among English language learners and medical students [2, 6]. Contrary to the present study, in the study of Sayadi and Soleimani, among English language students, academic enthusiasm is higher than the average [40]. During their studies, medical students face challenges and problems that can affect their academic enthusiasm and motivation. Care at the bedside and direct contact with many types of diseases is one of the things that can increase the difficulty of studying for these people and affect their academic enthusiasm. These reasons can be the possibility of the difference between the results of the above study and the present study. In a study in Saudi Arabia, Bayoumy and his colleagues reported that the level of academic motivation in nursing students is at a high level [41]. Although enthusiasm and passion may be slightly different from each other. However it should be considered that academic enthusiasm is a motivational construct, so the results of the two studies are not consistent. The difference in this study compared to our study can be justified considering the facilities available in the research location and the different structure of the educational system. On the other hand, the participants in this study were those who enrolled in the professional course of nursing education after completing the preliminary education course. Usually, the desire to study leads to the continuation of studies at higher levels and this justifies the difference in the results.

According to the results, the highest score in the academic enthusiasm tool was related to the field of “behavioral academic enthusiasm” which is in the average range. This finding is in line with some studies [42, 43]. In explaining the alignment between the findings of the aforementioned studies and the present research, it should be noted that an individual’s behaviors can be influenced by the cultural and contextual environment in which they live. Since both of the mentioned studies were also conducted in Iran and among university students, the consistency in results is justifiable. Although in Dorostkar Moghadam et al.’s study, the scores of this area are higher than the average, the highest average score is not related to this area [44]. In other words, the results of the two studies are not aligned. In interpreting the results, it should be noted that Dorostkar Moghadam et al.’s study was conducted among Azad University students, and in this type of university, there are various fields of humanities, engineering sciences, and medical sciences. Fields that exhibit significant differences in curriculum structure compared to medical sciences. Additionally, it should be noted that in Iranian Azad Universities, tuition fees are borne by the students themselves. This factor can significantly influence various variables.

According to the results, the field of “emotional academic enthusiasm” in these students was lower than the average level and was ranked second. Jafari et al. confirmed this finding in their study [7]. Contrary to the results of the present study, in Ahmadi et al.’s study, this area received the highest score [2]. Perhaps one of the reasons for the difference in the results can be attributed to the statistical population of the two studies. In Ahmadi et al.’s study, only medical students were included in the study, while the present study had a wider range of fields. The scores in this area in the intervention study of Aram et al. were higher than the average scores in the present study [45]. Considering that the mentioned study was conducted on English language learners, the results of this study cannot be generalized to students who study a wide range of subjects. On the other hand, the samples of the above study were students. According to the existing educational structure in Iran, entering a university means entering a larger social network, and this factor can affect students’ attitudes towards education and areas of academic enthusiasm.

The results of the present study showed that among the dimensions of academic enthusiasm, the lowest score is related to the field of cognitive academic enthusiasm. Although scores have not been standardized in many studies, in Ahmadi et al.’s study, this average range (13.4) was obtained similar to the present study [46]. Contrary to the results of the present study, Golshani et al., in a study on medical assistants, reported the average of this field to be higher than other fields [47]. Although Golshani et al. used a different tool to investigate academic enthusiasm, the difference in the results of this study with the present study can be due to the differences among the types of fields and the different views of the medical students of the residency level compared to other students in the lower levels.

### Loneliness

One of the important results of the present study was that the students of the present study have an average level of loneliness. In the study of Kupcewicz et al. in some European countries, the mean score of loneliness in nursing students is below average, which is in line with the findings of the present study [48]. Contrary to the results of the present study, in the study of Prasanna et al., students in India are at a mild level of loneliness [49]. The difference in results can be attributed to two points. First, the above study was conducted only among people with a bachelor’s degree, and second, it was conducted among male students with alopecia. In a study on Chinese students, most of the participants reported their loneliness level to be higher than the average level [50]. It seems that access to technology in advanced countries compared to developing countries has caused these students to spend more time using it. Also, the difference in the type of family structure in the Chinese study and our study can justify this.

### Fear of intimacy

This study showed that the fear of intimacy in students is lower than the average level. In a study conducted by Nabizadeh et al. among non-medical students, the students’ fear of intimacy scores (96.30) were somewhat close to the findings of the present study [51]. This finding is in line with the findings of two Iranian and Greek studies on adults [52, 53]. In the results of the study by Obeid et al. in Lebanon, it is stated that almost half of the participants are afraid of intimacy [54]. In fact, in the Lebanese study, the researchers considered the median score as the cut-off point of the tool and thus reported this finding; But by observing the results of this study, we find that this finding is in line with the results of the present study. Contrary to the results of the present study, in Lopez and Liu’s study, fear of intimacy is at a high level in college students [55]. The important point in interpreting fear of intimacy scores is that the lower the scores, the fewer problems a person has with intimacy. Therefore, it should be said that the students of the present study have fewer problems in establishing intimacy than in the above study.

### The role of fear of intimacy and loneliness in predicting of academic enthusiasm

One of the most important results of the present study was that the feeling of loneliness predicts the academic enthusiasm among medical science students, and the lower the feeling of loneliness in students, the greater the academic enthusiasm in this group. The feeling of loneliness is one of the crises of adolescence and youth that makes it difficult for a person to adapt to the environment. This feeling is a universal experience that occurs beyond age, culture, and social status [25]. The feeling of loneliness is very common among medical students [25]. According to the researcher, not only do these people enter a new environment, but they also experience the hospital environment’s mental and psychological pressures, the patient’s problems, and the large volume of lessons. Research indicates that increased interaction among students and teachers enhances enthusiasm [2]. In explaining this finding, it can be said that, when people feel more alone, they interact less with others, including teachers. This lack of interaction can negatively impact their academic enthusiasm. These observations align with the results of the present study. No study was found that examined exactly these two variables. However, Mizani et al.’s study among students showed that loneliness has an inverse and significant relationship with academic progress [21]. Also, in Hendrick et al.’s study, emotional loneliness predicting academic progress was reported [56]. Although the above studies did not consider academic enthusiasm, students who have more academic enthusiasm may experience academic progress in the future. Lin et al.’s study on Taiwanese students also showed that feeling lonely is correlated with a low sense of achievement [57]. In the qualitative study of Keykha and Nadi among students of medical sciences, the reduction of academic enthusiasm is one of the negative results of loneliness [58], which is in line with the findings of the present study. Another finding of the present study was that fear of intimacy does not play a significant role in predicting students’ academic enthusiasm. In the conducted searches, no similar study was found that addresses the relationship between fear of intimacy and academic enthusiasm. A study among medical science students revealed that fear of intimacy has a significant positive relationship with academic burnout and is capable of predicting academic burnout[38]. Although the aforementioned study did not consider academic enthusiasm, given that academic burnout and academic enthusiasm have an inverse relationship [59], it might be inferred that fear of intimacy indirectly affects academic enthusiasm through mediating variables. It appears that future studies should investigate the role of mediating variables in the relationship between fear of intimacy and academic enthusiasm.

### Limitations

The transferability of the results is one of the main limitations of the study due to cultural, social, and contextual differences. Another limitation of the study was the self-report form of data collection. Also, the psychological state of the participants while completing the questionnaire could have affected the results obtained. Therefore, efforts were made to ensure that the questionnaire was completed in a calm and stable environment.

## Conclusions

The findings of the present study showed that academic enthusiasm and feelings of loneliness are at an average level and fear of intimacy is below the average level. In addition, the results showed that the feeling of loneliness has a predictive role in academic enthusiasm so that as the feeling of loneliness increases, academic enthusiasm decreases. Given these results and the crucial role of medical science students in patient care, these students are expected to engage in cultural workshops, emotional management, communication skills, and academic motivation initiatives. So that we can see a decrease in the feeling of loneliness and an increase in academic enthusiasm. On the other hand, taking into account the importance and impact of loneliness on academic enthusiasm, managers and policymakers are expected to plan to improve mental health and academic progress in university students and create a suitable environment for students to express their emotions and feelings and to identify the most effective factors that create intimacy and their sense of loneliness. It is suggested that in these studies, the role of mediating variables in the relationship between the feeling of loneliness and the need to study should be investigated.

## Declarations

### Data availability

Data are available by contacting the corresponding author.
